# Supplementary material for: IL-6 receptor blockade corrects defects of XIAP-deficient regulatory T cells
Source: Nat Commun. 2018 Jan 31;9:463. doi: 10.1038/s41467-018-02862-4 (PMC5792625; doi:10.1038/s41467-018-02862-4)
Supplement: Supplementary file 1 — Supplementary Information [file 41467_2018_2862_MOESM1_ESM.pdf]

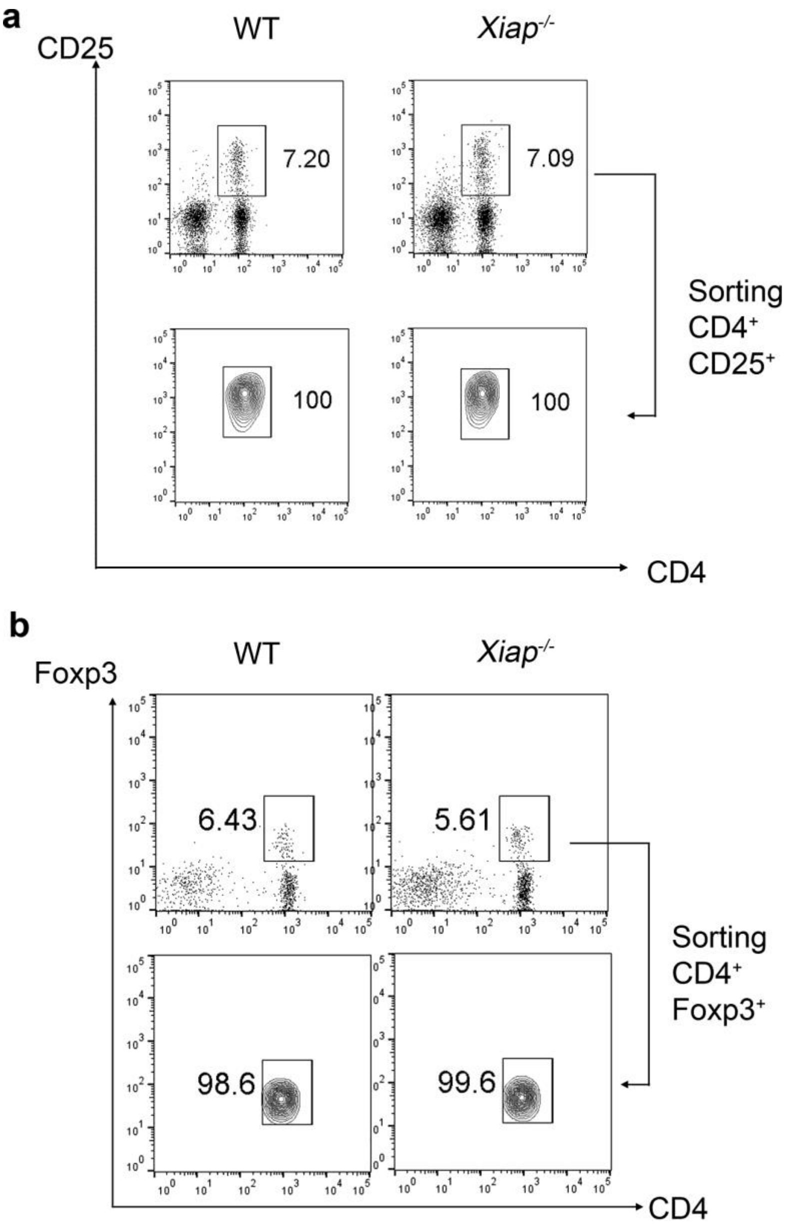

Supplementary Figure 1. The purity of mouse tTreg cells isolated by sorting. WT and *Xiap*$^{-/-}$ tTreg cells were isolated from splenocytes by sorting based on expression of CD4 and CD25. The purity of tTreg cells before and after sorting was assessed by CD4^{+} CD25^{+} (**a**) and CD4^{+} Foxp3^{+} (**b**).

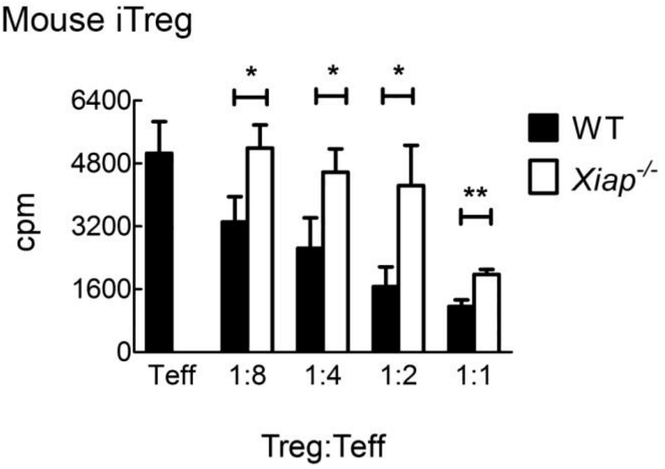

Supplementary Figure 2. Impaired suppressive activity of *Xiap*$^{-/-}$ iTreg cells. CD4^{+}CD25^{-} cells (1×10^{5}) were incubated with anti-CD3 (1 μg/ml), 3×10^{5} mitomycin C-treated T-depleted splenic cells, and the indicated ratios of WT and *Xiap*$^{-/-}$ iTreg cells. [^{3}H]thymidine incorporation was determined at 80 h. Values are mean ± SD of triplicate samples in an experiment. *$P < 0.05$, **$P < 0.01$ for unpaired t-test. Experiments were reproduced independently three times with similar outcomes.

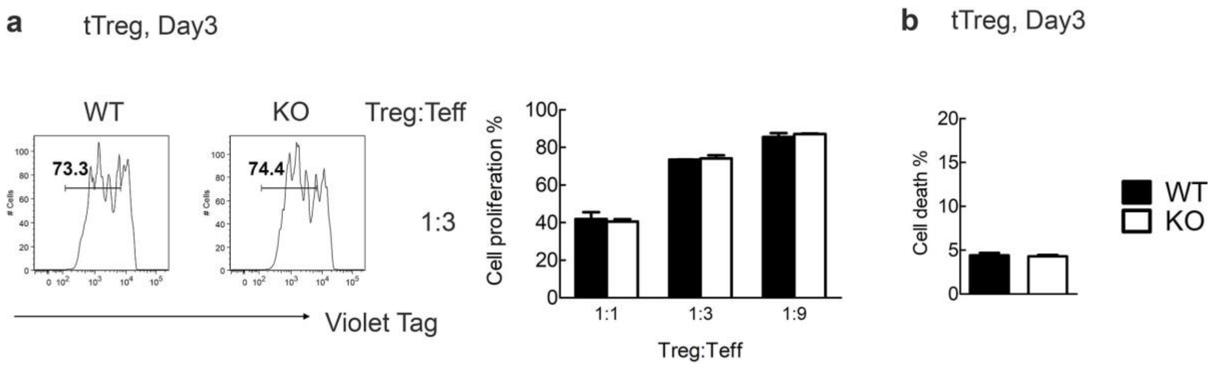

Supplementary Figure 3. Comparable proliferation and viability between WT and *Xiap*$^{-/-}$ tTreg cells. WT and *Xiap*$^{-/-}$ tTreg cells were labelled with CellTracker Violet, $CD4^{+}CD25^{-}$ (effector) T cells (at the indicated ratio) were labelled with CFSE, and were stimulated with anti-CD3 in the presence of mitomycin c-treated antigen-presenting cells for 3 days. tTreg cells were gated by CellTracker Violet, and the halving of CellTracker fluorescence was determined (**a**). The viability of the gated WT and *Xiap*$^{-/-}$ tTreg cells was also analyzed by simultaneous propium iodide staining (**b**).

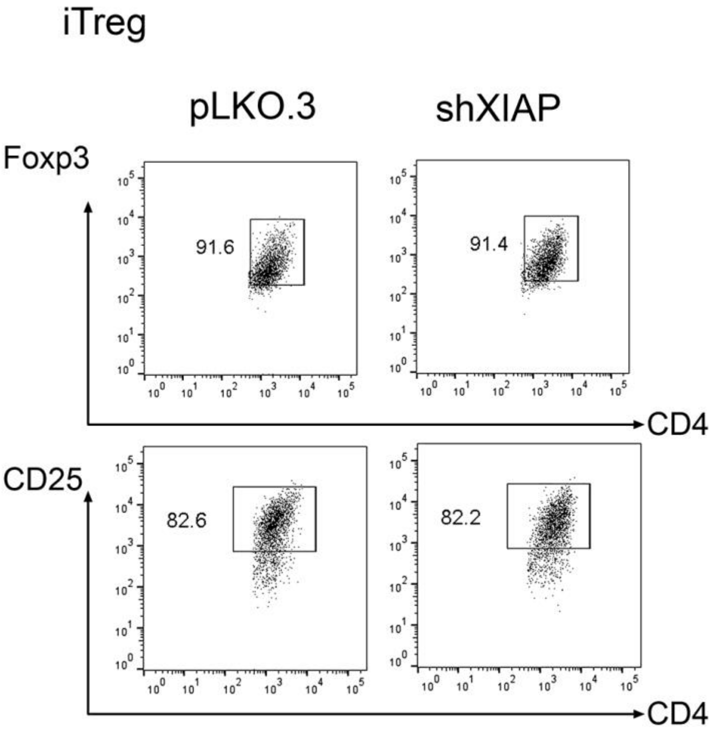

Supplementary Figure 4. XIAP knockdown does not affect human iTreg cell differentiation. XIAP was knocked down in naïve human CD4$^+$ T cells (CD4$^+$CD25$^-$CD45RA$^+$CD45RO$^-$) by control (pLKO.3) or pLKO.3-shXIAP lentiviral infection, and the infected T cells were isolated after 4 days. T cells were rested for 8 days and re-isolated. Control and XIAP-knockdown naïve CD4$^+$ T cells were differentiated into iTreg cells with immobilized anti-CD3/CD28 in the presence of IL-2 (20 ng/ml) and TGF-β (5 ng/ml) for 5 days. The levels of CD25 and Foxp3 expression in control (pLKO.3) and XIAP-knockdown iTreg cells were determined. The experiments were independently repeated three times with similar results.

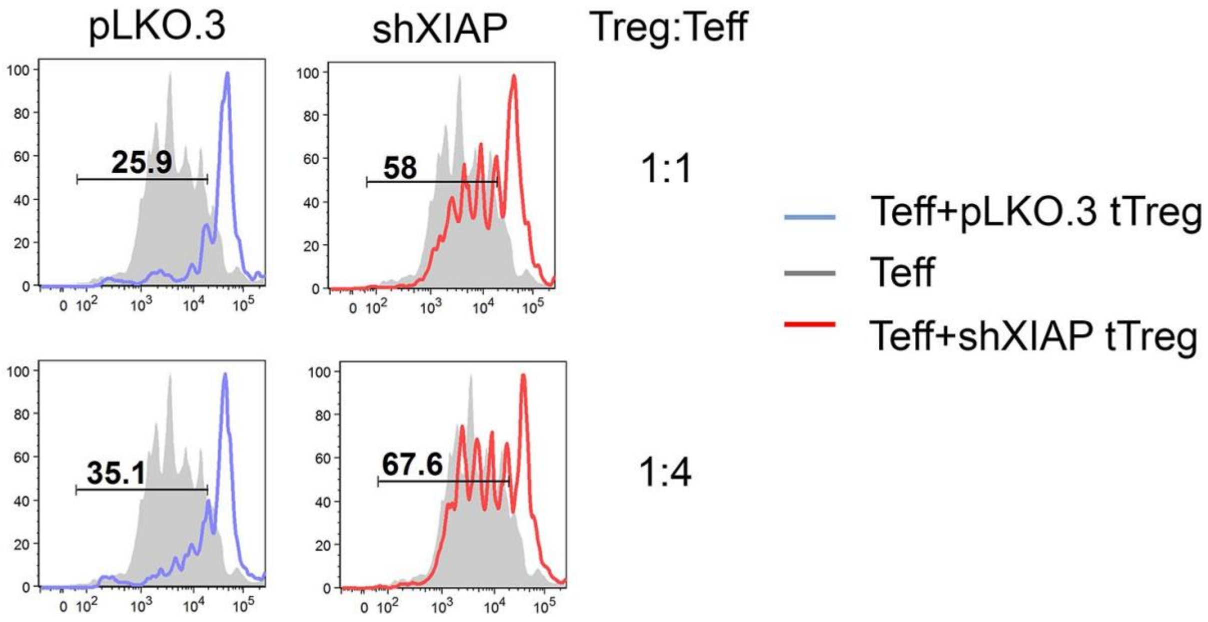

Supplementary Figure 5. Impaired suppressive activities of human tTreg cells as measured by CFSE halving. Human T cell effector cells ($CD4^{+}CD25^{-}$) were labeled with 2 μM CFSE, and were then incubated with different ratios of control (pLKO.3) and XIAP-knockdown human tTreg cells in the presence of anti-CD3 and mitomycin C-treated dendritic cells for 72 h. $CD4^{+}$ T cells were gated for CFSE intensity determination. Numbers indicate the fraction of proliferating T cells (i.e. with CFSE halving). Grey-shadowed curve represents proliferation of Teff in the absence of tTreg cells.

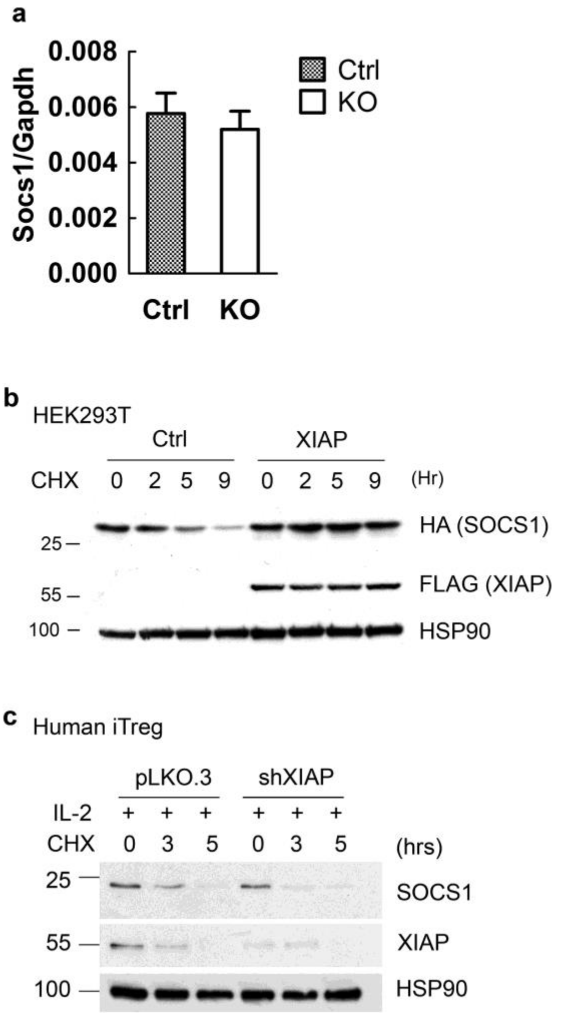

Supplementary Figure 6. XIAP increases protein stability of SOCS1. (**a**) Normal *Socs1* transcript levels in *Xiap*$^{-/-}$ T cells. The quantities of *Socs1* mRNA were determined in WT and *Xiap*$^{-/-}$ T cells by quantitative PCR. (**b**) XIAP increases the stability of SOCS1 protein. HEK293T cells were transfected with SOCS1 with or without XIAP. Forty-eight hours later, cells were treated with cycloheximide (CHX, 10 ng ml^{-1}), and the levels of SOCS1 and XIAP at the indicated time-points were determined. (**c**) XIAP-knockdown decreases the stability of SOCS1 protein in human iTreg cells. Human iTreg cells (1x10^{6}) were pre-treated with hIL-2 (100 ng ml^{-1}) for 30 mins, followed by treatment with CHX (20 μg ml^{-1}), and the levels of SOCS1 and XIAP at the indicated time-points were determined. The experiments (a, b) were independently repeated three times with similar results.

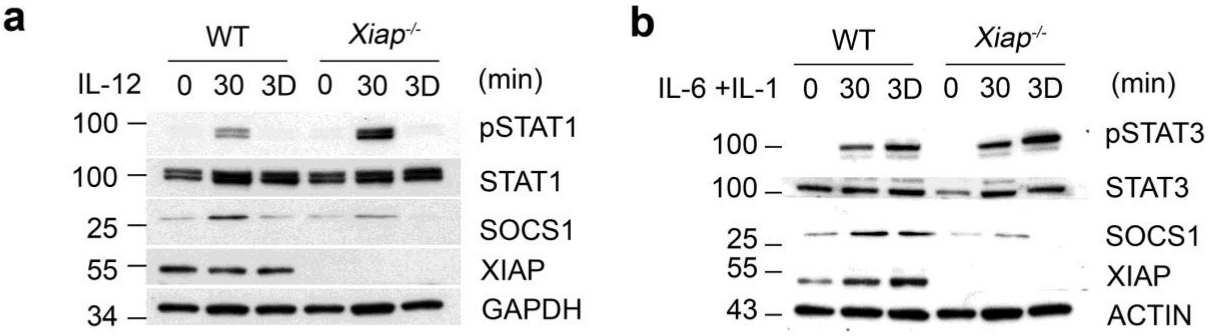

Supplementary Figure 7. Enhanced activation by IL-12 and IL-6 stimulation in *Xiap*$^{-/-}$ iTreg cells. (**a**) Increased STAT1 phosphorylation in IL-12-treated *Xiap*$^{-/-}$ iTreg cells. Fully differentiated WT and *Xiap*$^{-/-}$ iTreg cells were rested for 3 days, followed by re-stimulation with IL-12 (50 ng ml^{-1}) for 30 min and 3 days (3D). The extent of STAT1 phosphorylation was revealed by immunoblot. (**b**) Enhanced STAT3 phosphorylation in *Xiap*$^{-/-}$ iTreg cells stimulated with IL-6 plus IL-1. WT and *Xiap*$^{-/-}$ iTreg cells were treated with IL-1 (IL-1α and IL-1β, 20 ng ml^{-1} each) plus IL-6 (50 ng ml^{-1} each) for 30 min and 3 days (3D), and the level of STAT3 phosphorylation was revealed by immunoblot. Experiments (a, b) were independently repeated three times with comparable results.

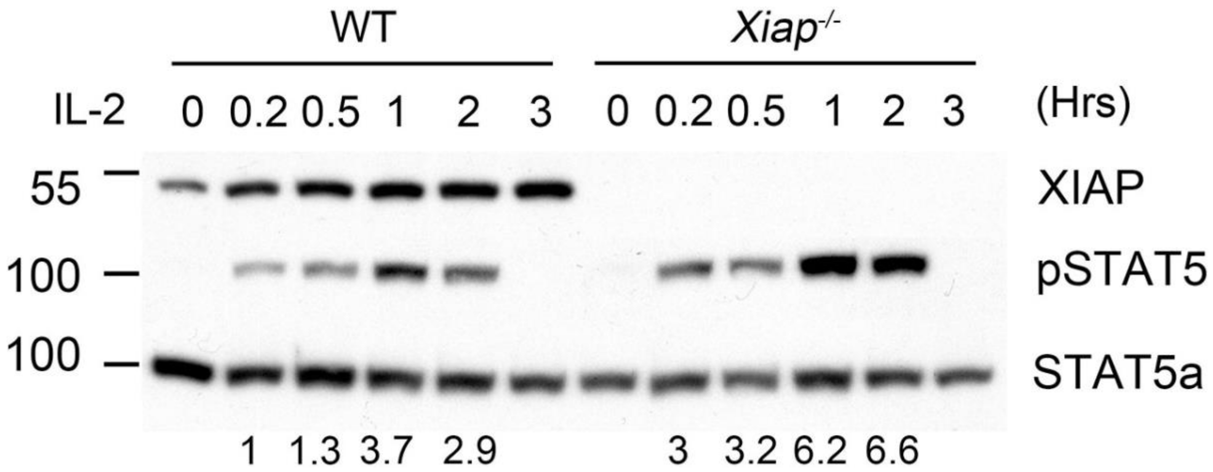

Supplementary Figure 8. Increased IL-2-induced STAT5 phosphorylation in *Xiap*$^{-/-}$ T cells. WT and *Xiap*$^{-/-}$ T cells were treated with IL-2, cell lysates were isolated at the indicated time-points, and the levels of phosphorylated STAT5 and STAT5 were revealed by immunoblots. The experiment was independently repeated three times with comparable results.

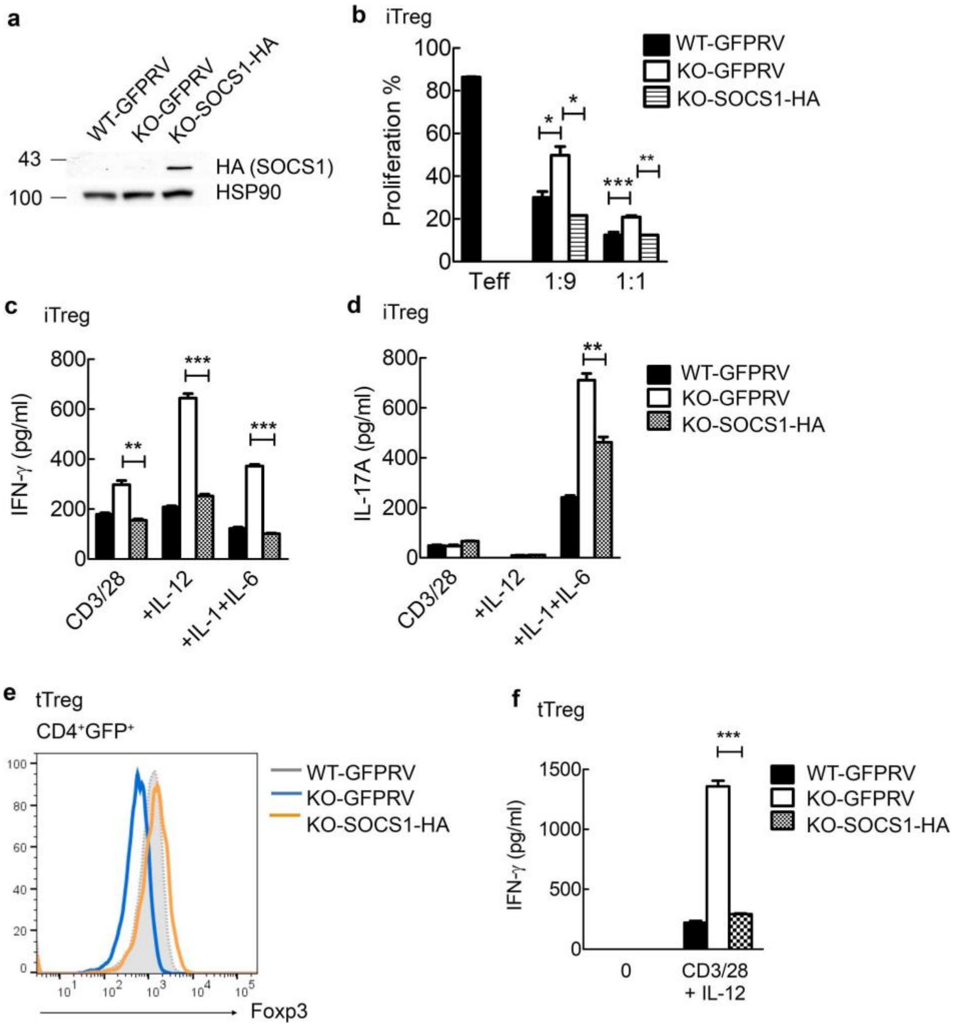

Supplementary Figure 9. Re-introduction of SOCS1 stabilizes *Xiap*$^{-/-}$ Treg cells. (**a**) Overexpression SOCS1-HA in differentiated iTreg cells. GFPRV and GFPRV-SOCS1-HA were used to infect WT or *Xiap*$^{-/-}$ iTreg cells. GFP$^+$CD25$^+$ cells were isolated 3 days later by sorting. SOCS1 expression was determined by anti-HA. (**b**) Overexpressed SOCS1 restores the suppressive activity in *Xiap*$^{-/-}$ iTreg cells. T effector cells (Teff) were labeled with 2 μM Violet-Tag, and activated by antigen-presenting cells and anti-CD3 in the presence of the indicated ratio of WT-GFPRV, KO-GFPRV or KO-GFPRV-SOCS1 iTreg cells. Proliferation of Teff was determined 72 h later by quantitation of Violet-Tag intensity. (**c, d**) Re-introduction of SOCS1 prevents IFN-γ and IL-17A production in *Xiap*$^{-/-}$ iTreg cells stimulated by IL-12 or IL-1/IL-6. WT-GFPRV, KO-GFPRV and KO-GFPRV-SOCS1 iTreg cells were stimulated with anti-CD3/CD28 and IL-2, in the absence or presence of IL-12 or IL-1 + IL-6, for 4 days. The production of IFN-γ (c) and IL-17A (d) was determined after re-activation by TPA/23187. (**e**) Overexpression of SOCS1 increases Foxp3 stability and inhibits IFN-γ generation in *Xiap*$^{-/-}$ tTreg cells. WT-GFPRV, KO-GFPRV and KO-GFPRV-SOCS1-HA tTreg cells were stimulated with anti-CD3/CD28, IL-2 and IL-12 for 5 days. tTreg cells were re-stimulated with TPA/A23187 for 5 h and Foxp3 expression was determined (e), or tTreg cells were re-stimulated with TPA/A23187 for 24 h and IFN-γ production was quantitated (f). Values (b, c, d, f) are mean ± SD of triplicate samples in an experiment. $*P < 0.05$, $**P < 0.01$, $***P < 0.001$ for unpaired t-test. All experiments were independently repeated three times with similar results.

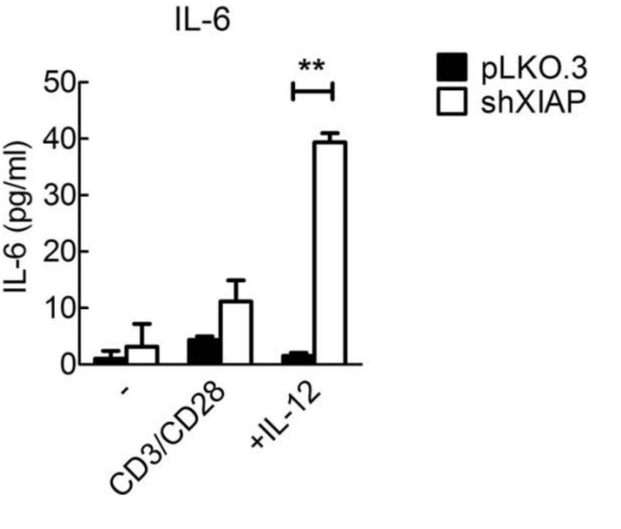

Supplementary Figure 10. Increased secretion of IL-6 by human XIAP-knockdown iTreg cells. Control and human XIAP-knockdown iTreg cells were activated by anti-CD3/CD28 and IL-2, with the addition of IL-12, as indicated, for 4 days. Secretion of IL-6 was quantitated by ELISA after re-stimulation with TPA/A23187 for 24 h. Values are mean ± SD of triplicate samples in an experiment. $^{**}P < 0.01$ for unpaired t-test. The experiment was independently repeated three times with similar results.

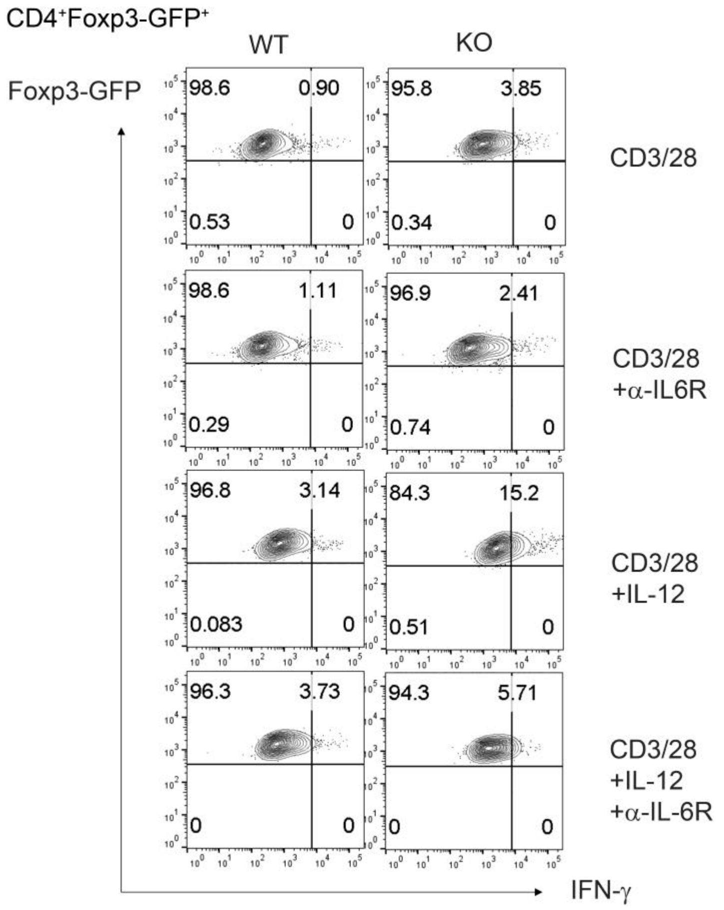

Supplementary Figure 11. Anti-IL-6R reduces the expression of IFN-γ in activated Foxp3-GFP-tagged *Xiap*$^{-/-}$ tTreg cells. Foxp3-GFP-tagged WT and *Xiap*$^{-/-}$ tTreg cells (as in Fig. 5a) were stimulated with anti-CD3/CD28 and IL-2, and with IL-12 as indicated, in the absence or presence of anti-IL-6R (50 µg ml^{-1}) for 4 days. tTreg cells were reactivated with TPA/A23187 for 5 h and the GFP^{+} (representing Foxp3^{+}) fraction was gated as in Fig. 5a before IFN-γ expression was determined by intracellular staining. The experiment was independently repeated three times with similar results.

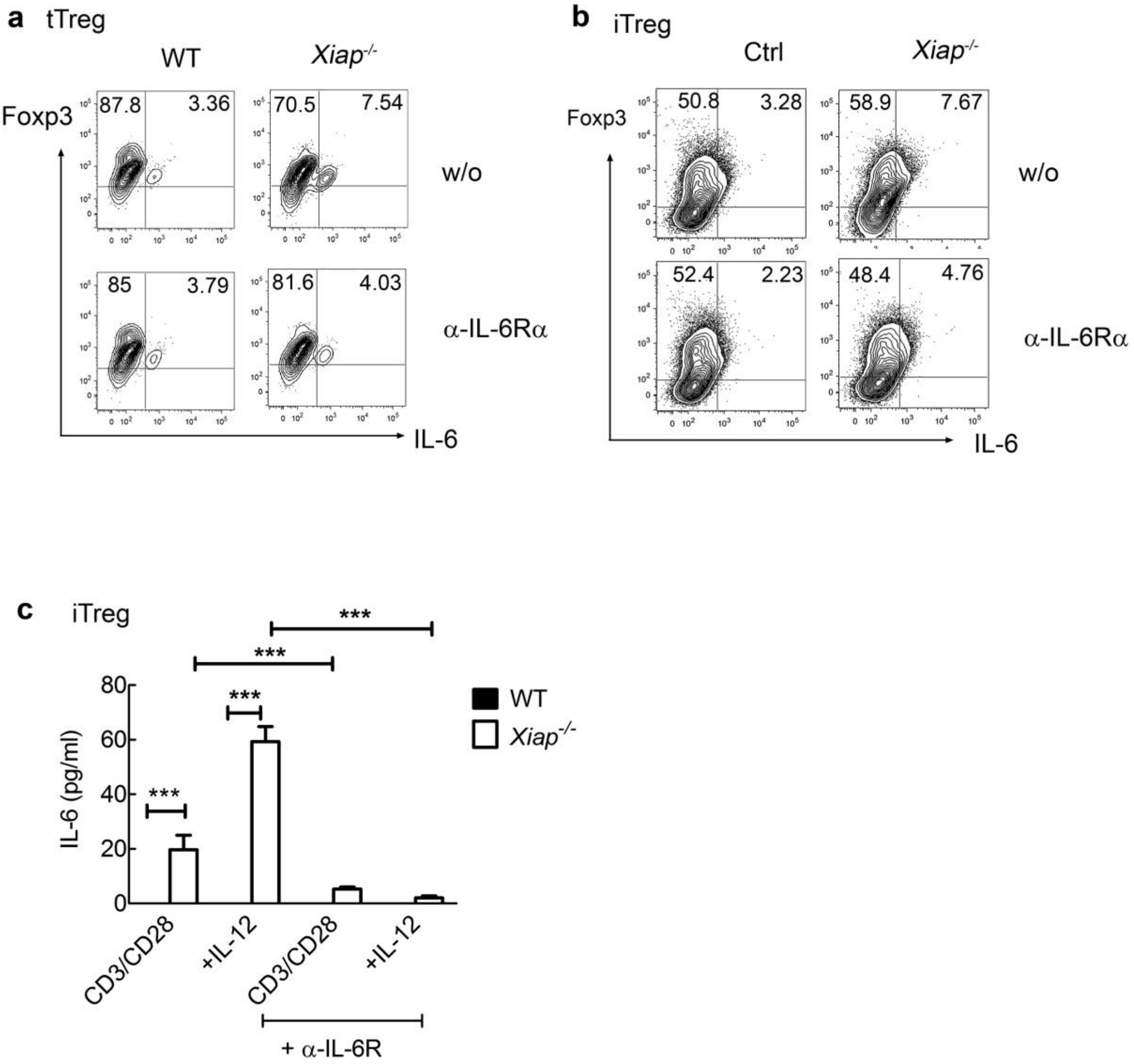

Supplementary Figure 12. Anti-IL-6R reduces the expression of IL-6 in activated *Xiap*$^{-/-}$ Treg cells. WT and *Xiap*$^{-/-}$ tTreg cells (**a**) or iTreg cells (**b**) were stimulated with anti-CD3/CD28 and IL-2, with or without IL-12, in the absence or presence of anti-IL-6R (50 μg/ml) for 4 days. Treg cells were reactivated with TPA/A23187 for 5 h and the expression of Foxp3 and IL-6 were determined by staining. iTreg cells were also re-stimulated with TPA/A23187 for 24 h and IL-6 production was determined by ELISA (**c**). Values are mean ± SD of triplicate samples in an experiment. ***$P < 0.001$ for unpaired t-test. The experiments (a-c) were independently repeated three times with similar results.

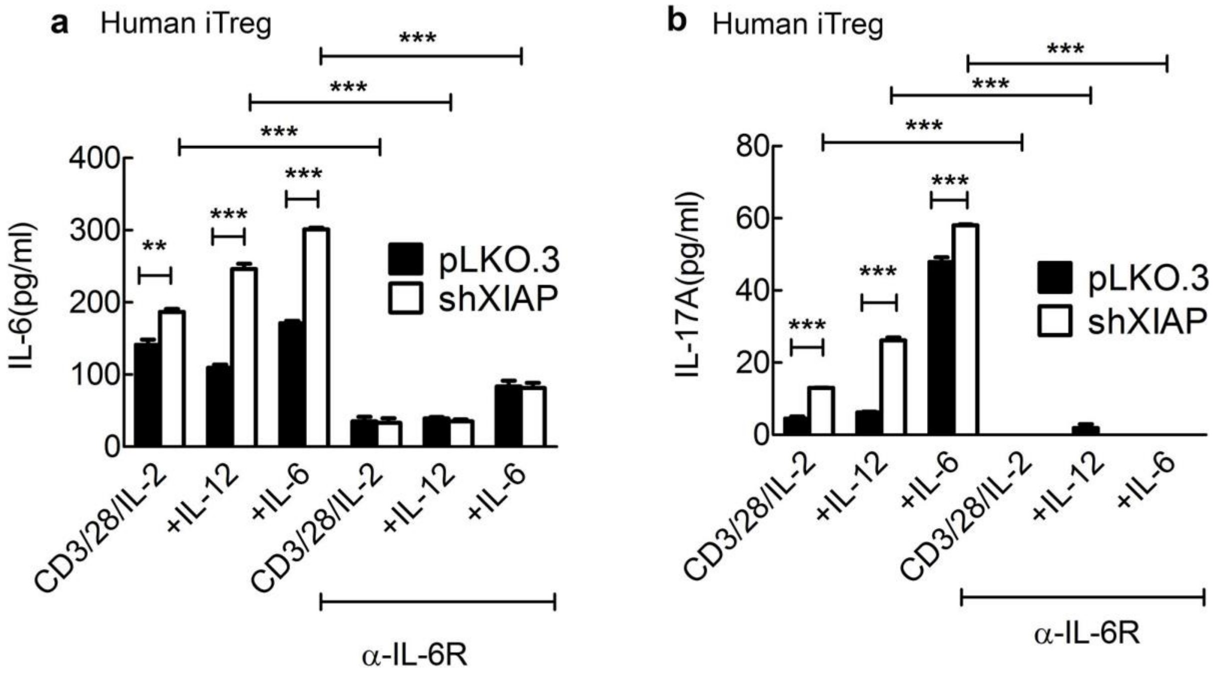

Supplementary Figure 13. Anti-IL-6R suppresses the production of IL-17 or IL-6 in activated human iTreg cells. Control (pLKO.3) and human XIAP-knockdown (shXIAP) iTreg cells were stimulated with anti-CD3/CD28 and IL-2, with the addition of IL-12, IL-6 or anti-IL-6R, as indicated, for 4 days. Secretion of IL-17 (**a**) and IL-6 (**b**) was quantitated by ELISA after re-stimulation with TPA/A23187 for 24 h. Values are mean ± SD of triplicate samples in an experiment. $^{**}P < 0.01$, $^{***}P < 0.001$ for unpaired t-test. The experiments (a, b) were independently repeated three times with similar results.

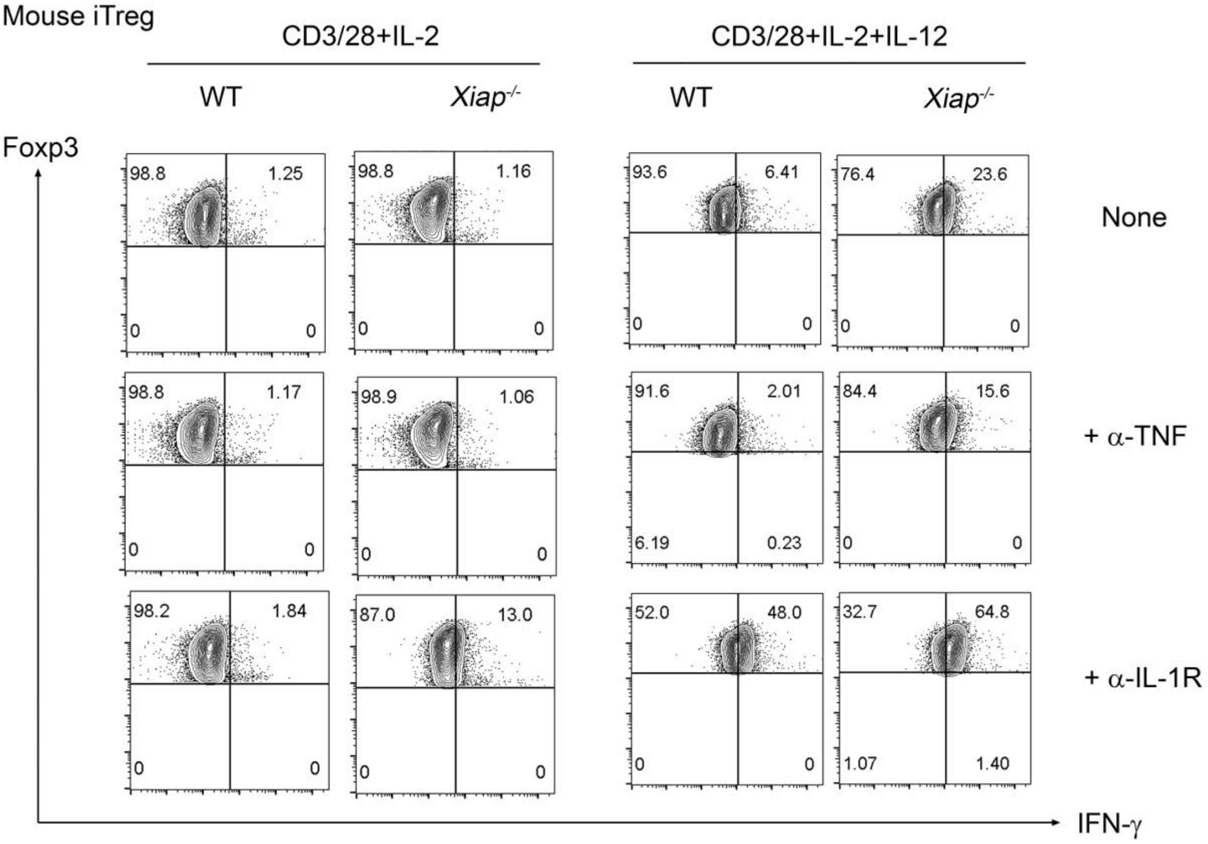

Supplementary Figure 14. Inability of anti-TNF or anti-IL-1R to inhibit the expression of IFN-γ in activated *Xiap$^{-/-}$* Treg cells. WT and *Xiap$^{-/-}$* iTreg cells were stimulated with anti-CD3/CD28 and IL-2, with or without IL-12, in the presence or absence of anti-TNF or anti-IL-1R (50 μg ml^{-1}each) for 4 days. Treg cells were reactivated with TPA/A23187 for 6 h and the expressions of Foxp3 and IFN-γ were determined by staining. The experiments were independently repeated three times with similar results.

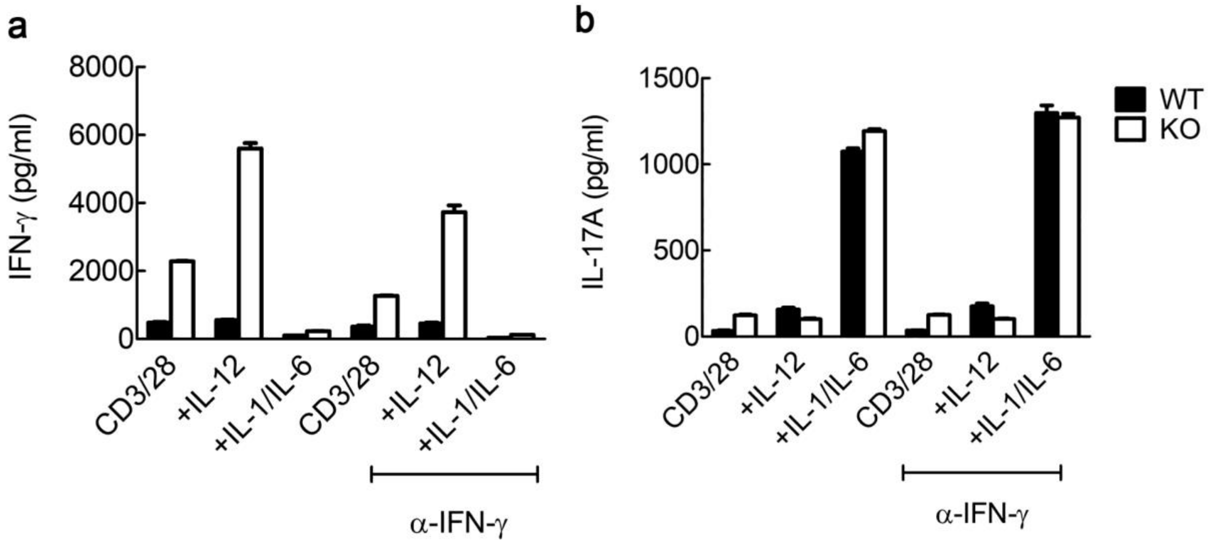

Supplementary Figure 15. Infectiveness of anti-IFN-γ to inhibit IL-12-promoted expression of IFN-γ and IL-17 in activated *Xiap*$^{-/-}$ Treg cells. WT and *Xiap*$^{-/-}$ iTreg cells were stimulated with anti-CD3/CD28 and IL-2, with or without IL-12, in the presence or absence of anti-IFN-γ (50 μg ml^{-1} each) for 4 days. Treg cells were reactivated with TPA/A23187 for 24 h and the production of IFN-γ (**a**) and IL-17A (**b**) were determined by ELISA. Values are mean ± SD of triplicate samples in an experiment. Experiments were independently repeated three times with similar results.

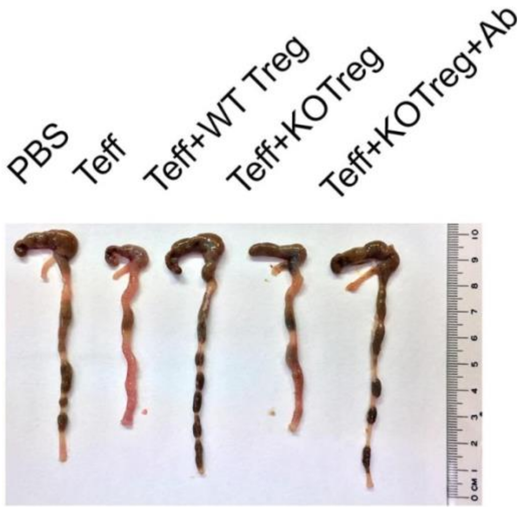

Supplementary Figure 16. Anti-IL-6R restores the suppressive activity of *Xiap*$^{-/-}$ tTreg cells *in vivo.* CD45.2^{+} WT or *Xiap*$^{-/-}$ tTreg cells were co-transferred with CD45.1^{+} CD4^{+}CD25^{-} effector T cells into CD45.1^{+} *RagI*$^{-/-}$ mice. Anti-IL-6R antibody (500 μg/mouse) was intraperitoneally administrated at day 0, followed by weekly dosing of 500 μg. Mice were sacrificed at day 27 and the morphology of their colons was examined.

Fig. 1e

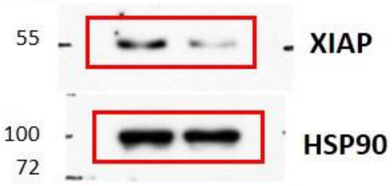

Fig. 3a

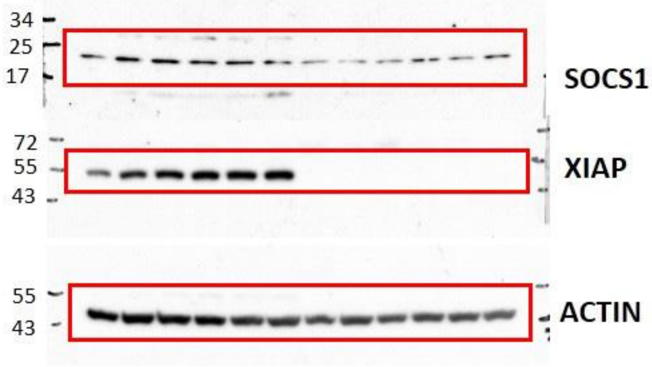

Fig. 3b

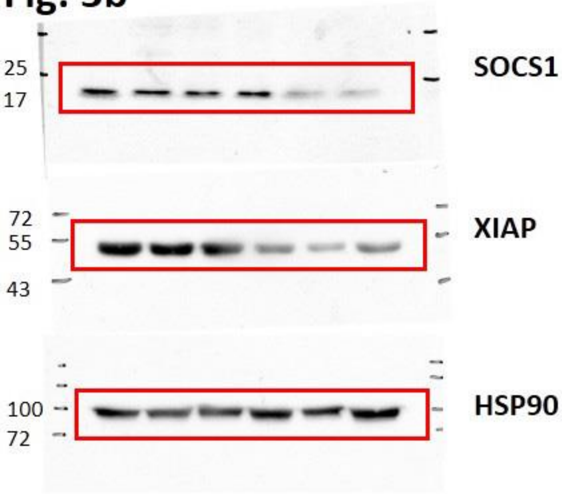

Fig. 3c

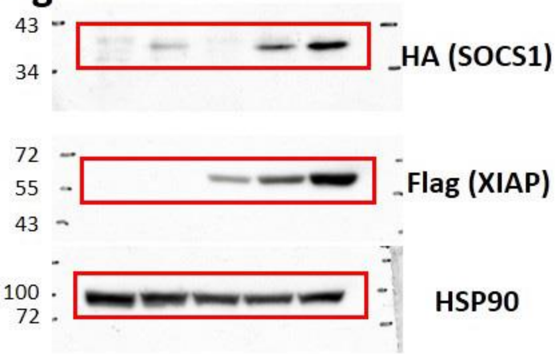

Fig. 3d

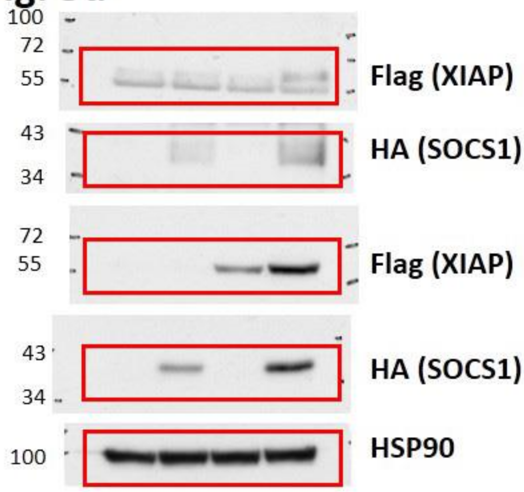

Fig. 3e

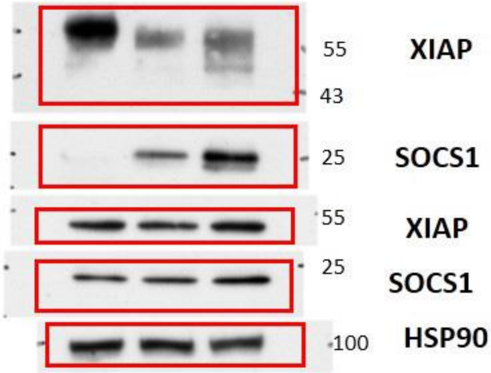

Supplementary Figure 17. Uncropped images of the original scans of immunoblots. Uncropped, full-size scans of immunoblots shown in Fig. 1e, 3a, 3b, 3c, 3d and 3e.

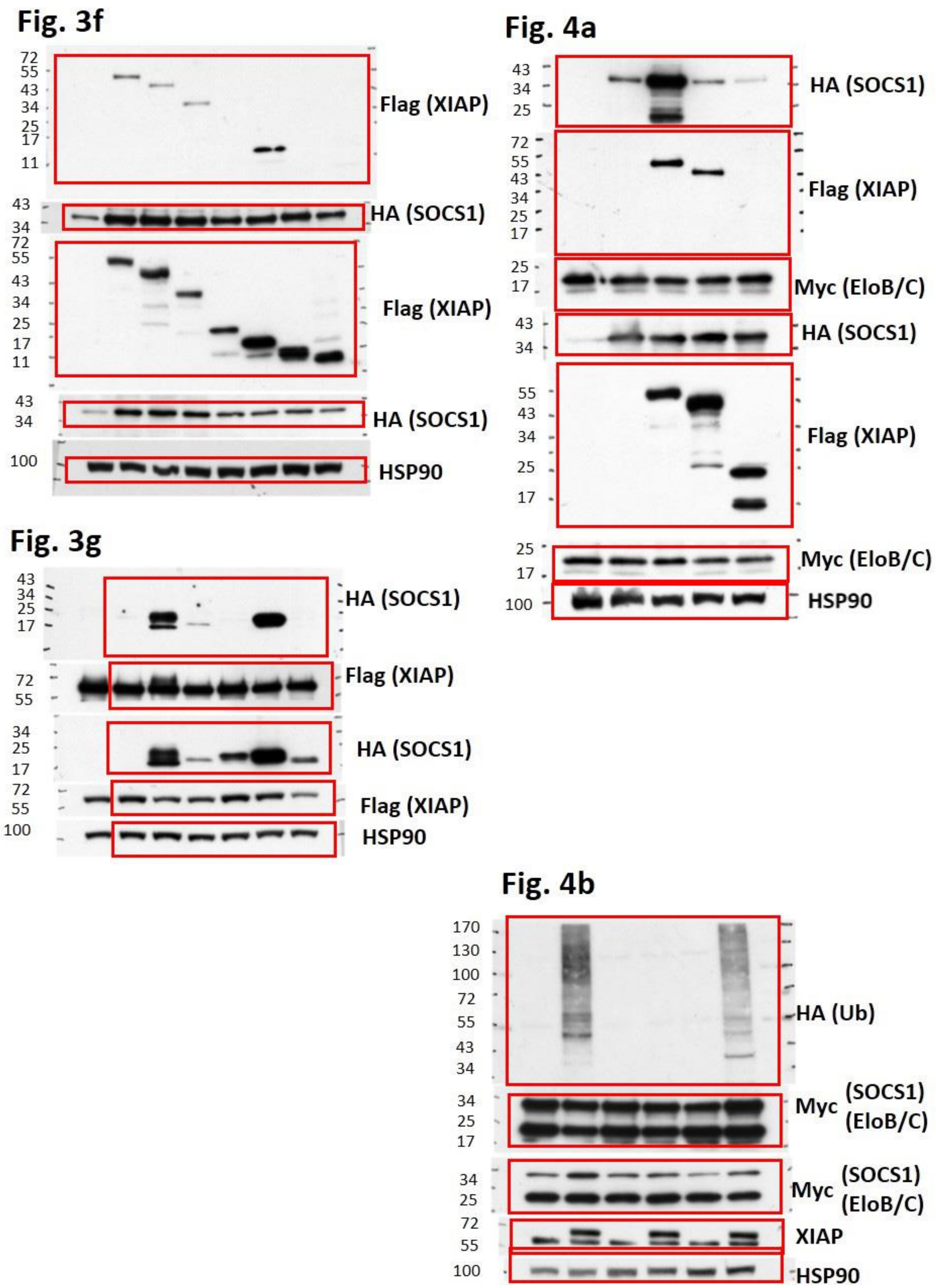

Supplementary Figure 18. Uncropped images of the original scans of immunoblots.
Uncropped, full-size scans of immunoblots shown in Fig. 3f, 3g, 4a, and 4b.

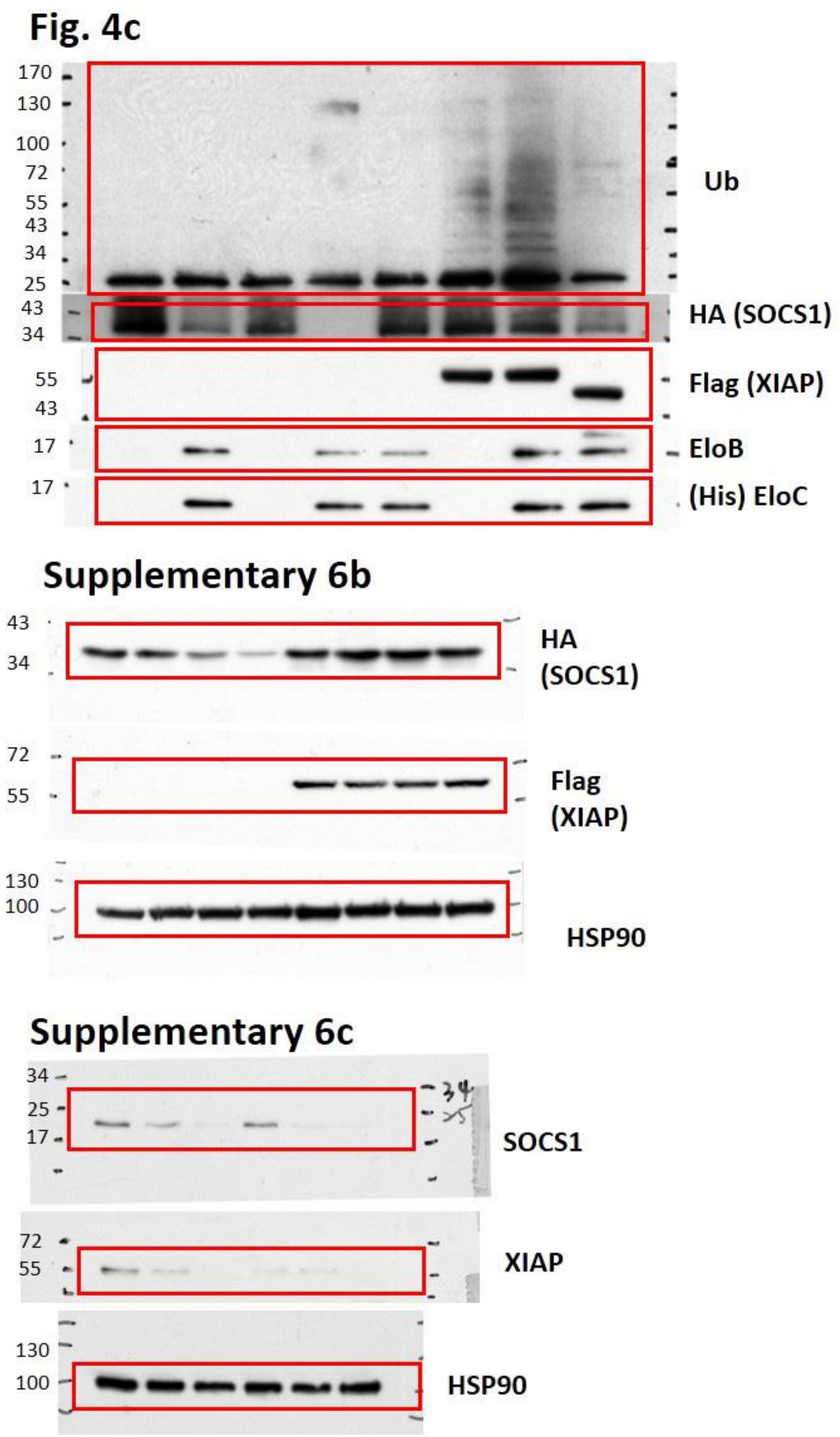

Supplementary Figure 19. Uncropped images of the original scans of immunoblots. Uncropped, full-size scans of immunoblots shown in Fig. 4c, Supplementary Fig. 6b and 6c.

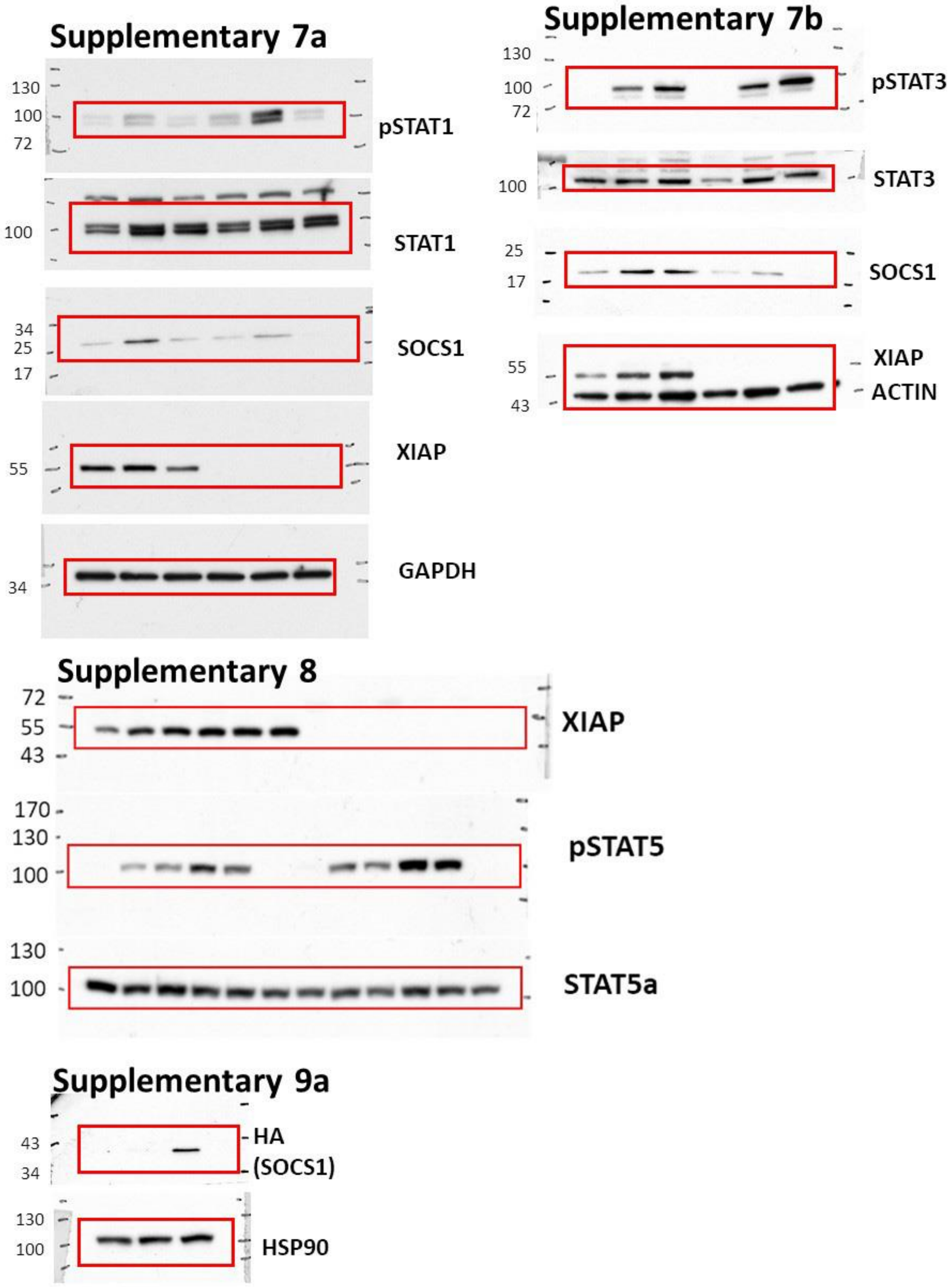

Supplementary Figure 20. Uncropped images of the original scans of immunoblots. Uncropped, full-size scans of immunoblots shown in Supplementary Fig. 7a, 7b, 8, and 9a.

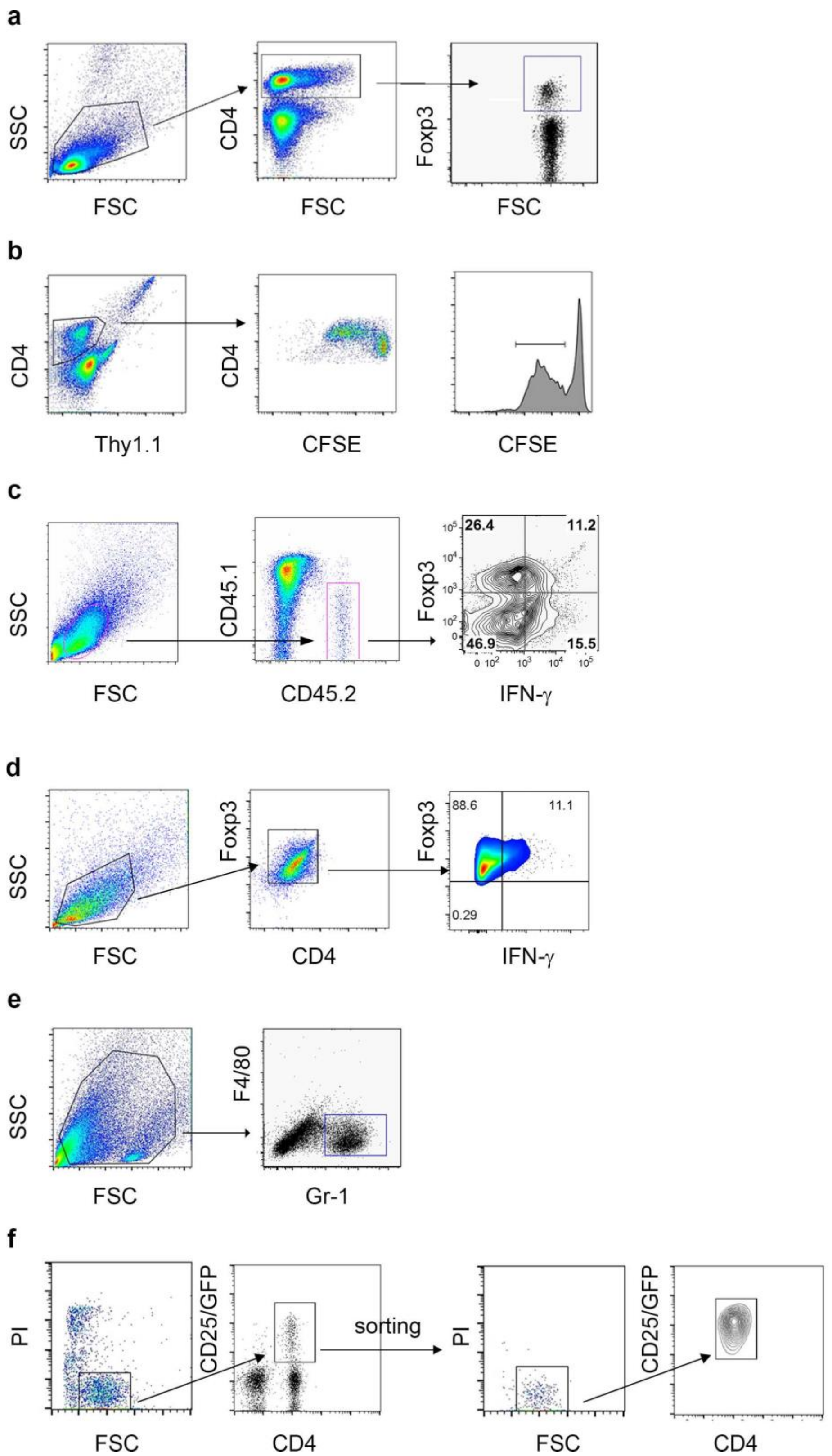

Supplementary Figure 21. FACS gating/sorting strategies. (a) FACS gating strategies used in Fig. 1a, 1b, and 2a. **(b)** FACS gating strategies used in Fig. 1f and Supplementary Fig. 5. **(c)** FACS gating strategies used in Fig. 2c, 5g, 5h, 7g, 7h, and 8c. **(d)** FACS gating strategies used in Fig. 5e, 7b, and 7d. **(e)** FACS gating strategies used in Fig. 6c. **(f)** FACS sorting strategies used in Supplementary Fig. 1 and all Treg cells sorting in this study.

Supplementary Table 1
Sequences of primers for plasmid construction

hXIAP-N-terminal (1-333 aa) fragment	5’-GCG GGA TCC AGA TGA CTT TTA ACA GTT TTG AAG-3’<br>5’- GCG CTC GAG CTG TTC TAA CAG ATA TTT G-3’
hXIAP-C-terminal (330-498 aa) fragment	5’- GCG GAA TTC ATG AGG GAC AAG AAT ATA TAA ACA A-3’<br>5’- GCG CTC GAG AGA CAT AAA AAT TTT TTG CTT GAA AG-3’
hXIAP-BIR1 (1-131 aa) fragment	5’- GCG GGA TCC AGA TGA CTT TTA ACA GTT TTG AAG-3’<br>5’- GCG CTC GAG AGA TGG CCT GTC TAA GGC AAA-3’
hXIAP-BIR2 (131-233 aa) fragment	5’- GCG GGA TCC ATG ACA CAT GCA GAC TAT CTT TT-3’<br>5’- GCG CTC GAG CCG GCC CAA AAC AAA GAA GC-3’
hXIAP-BIR3 (261-333 aa) fragment	5’- GCG GGA TCC ATG GCA GAT TAT GAA GCA C-3’ and 5’- GCG CTC GAG CTG TTC TAA CAG ATA TTT G-3’.
Human Elongin B	5’-GCG GGA TCC CGA TGG ACG TGT TCC TCA TGA TCC GG<br>5’-GCG CTC GAG CTG CAC GGC TTG TTC ATT GGC ACT GC.
Human Elongin C	5’- GCG GGA TCC AAA TGG ATG GAG AGG AGA AAA CCT ATG G-3’<br>5’-GCG CTC GAG ACA ATC TAA GAA GTT CGC AGC CAT CAG-3’.
Full-length hSOCS1	5’- GCG GGA TCC ACA TGG TA G CAC ACA ACC AGG TGG C-3’<br>5’- GCG CTC GAG AAT CTG GAA GGG GAA GG A GCT CAG GT-3’.
hSOCS1-ΔSB (1-174 aa)	5’- GCG GGA TCC GGA TGG TA G CAC ACA ACC AGG TGG CA-3’<br>5’- GCG CTC GAG CGC CGC CAC GTA GTG CTC CAG-3’
hSOCS1-ΔN-terminal (78-211 aa)	5’-GCG GGA TCC GG ATG TT C TAC TGG GGG CCC CTG AG-3’ and 5’- GCG CTC GAG AAT CTG GAA GGG GAA GGA GCT CAG-3’
hSOCS1-N-terminal (1-77 aa)	5’-GCG GGA TCC ACA TGG TA G CAC ACA ACC AGG TGG C-3’<br>5’-GCG CTC GAG TCC GCA GGC GTC CAG GAG CG-3’
hSOCS1-SH2 (78-174 aa)	5’-GCG GGA TCC GG ATG TT C TAC TGG GGG CCC CTG AG-3’<br>5’-GCG CTC GAG CAG CGG CCG CAC GCG GCG C-3’
hSOCS1-SB (170-211 aa)	5’-GCG GGA TCC GGA TGG TGC GGC CGC TGC AGG AG-3’ and 5’- GCG CTC GAG AAT CTG GAA GGG GAA GG A GCT CAG GT-3’
